# A qualitative study of transgender individuals’ experiences in residential addiction treatment settings: stigma and inclusivity

**DOI:** 10.1186/s13011-015-0015-4

**Published:** 2015-05-07

**Authors:** Tara Lyons, Kate Shannon, Leslie Pierre, Will Small, Andrea Krüsi, Thomas Kerr

**Affiliations:** British Columbia Centre for Excellence in HIV/AIDS, St. Paul’s Hospital, 608-1081 Burrard Street, Vancouver, BC V6Z 1Y6 Canada; Department of Medicine, University of British Columbia, 317-2194 Health Sciences Mall, Vancouver, BC V6T 1Z3 Canada; School of Population and Public Health, University of British Columbia, 5804 Fairview Avenue, Vancouver, BC V6T 1Z3 Canada; Providing Alternatives Counselling & Education Society (PACE), 49 W. Cordova St, Vancouver, BC V6B 1C8 Canada; Faculty of Health Sciences, Simon Fraser University, 8888 University Drive, Burnaby, BC V5A 1S6 Canada

**Keywords:** Transgender, Treatment, Enacted stigma, Felt stigma, Inclusion, Indigenous, Drug use

## Abstract

**Background:**

While considerable research has been undertaken on addiction treatment, the experiences of transgender individuals who use drugs are rarely explored in such research, as too often transgender individuals are excluded entirely or grouped with those of sexual minority groups. Consequently, little is known about the treatment experiences in this population. Thus, we sought to qualitatively investigate the residential addiction treatment experiences of transgender individuals who use illicit drugs in a Canadian setting.

**Methods:**

In-depth semi-structured interviews were conducted with 34 transgender individuals in Vancouver, Canada between June 2012 and May 2013. Participants were recruited from three open prospective cohorts of individuals who use drugs and an open prospective cohort of sex workers. Theory-driven and data-driven approaches were used to analyze the data and two transgender researcher assistants aided with the coding and the interpretation of data in a process called participatory analysis.

**Results:**

Fourteen participants had previous experience of addiction treatment and their experiences varied according to whether their gender identity was accepted in the treatment programs. Three themes emerged from the data that characterized individuals’ experiences in treatment settings: (1) enacted stigma in the forms of social rejection and violence, (2) transphobia and felt stigma, and (3) “trans friendly” and inclusive treatment. Participants who reported felt and enacted stigma, including violence, left treatment prematurely after isolation and conflicts. In contrast, participants who felt included and respected in treatment settings reported positive treatment experiences.

**Conclusions:**

The study findings demonstrate the importance of fostering respect and inclusivity of gender diverse individuals in residential treatment settings. These findings illustrate the need for gender-based, anti-stigma policies and programs to be established within existing addiction treatment programs. Additionally, it is vital to establish transgender and/or LGBTQ specific treatment programs as recommended by the participants in this study.

## Background

Although the body of research focused on addiction treatment processes and outcomes has continued to grow, transgender individuals who use drugs have typically been excluded from such research, or they have been grouped with those of sexual minority and/or cisgender (individuals whose assigned sex corresponds to their gender identity and gender expression [1]) groups. Thus, treatment experiences among transgender persons have not been well documented and the results to date are mixed. While high rates of substance use have been documented among some transgender populations [2,3], other studies have found scant differences in substance use patterns among transgender and cisgender groups [4]. Transgender women have been found to be more likely to report syringe use; however, it has not been established whether this is indicative of the injection of hormones and/or substance use [4,5]. Further, while rates of substance use among transgender groups are not concrete, transgender persons have reported difficulty accessing addiction treatment programs [6] and healthcare more broadly [7].

Barriers to addiction treatment for transgender persons are often rooted in stigma and include structural barriers (e.g., sex segregated housing) as well as treatment provider attitudes. Past research suggests that many treatment professionals report stigmatizing attitudes towards their LGBTQ clients and also lack knowledge of LGBTQ-related issues [8,9]. Further, treatment providers working with LGBTQ individuals receive little if any education into the specific treatment needs of gender and sexual minorities [10,11]. For example, in a study comparing rural and urban treatment providers in the US, Eliason and Hughes [9] found an average of 1 hour of training on transgender issues among counselors in the rural setting, and 2.4 hours in the urban setting. With limited training and understanding of transgender populations, treatment providers may contribute to barriers to addiction treatment, including stigmatizing attitudes.

Stigmas acts as a barrier to health services, including addiction treatment, and it is understood as a process by which marginalized individuals or groups are labeled with negative, often stereotypical, characteristics which contribute to harmful outcomes (e.g., social exclusion) [12]. Stigmatization is a social process dependent upon power that nurtures and reproduces social inequalities; consequently there are multiple ways stigma occurs [12-14]. For example, enacted stigma is characterized as incidents of discrimination (e.g., rejection, violence) and felt stigma refers to an internalization of stigma which manifests as the fear of experiencing some form of enacted stigma [15]. Therefore, there may be complexities surrounding experiences of stigma for transgender persons in addiction treatment settings.

While there is some literature on substance use among transgender groups and treatment provider training, studies investigating transgender individuals experience with addiction treatment are scarce. Transgender participants in a New York study reported lower satisfaction with treatment and lower rates of abstinence and treatment completion compared to heterosexual, gay and bisexual counterparts [16]. In a study of transgender indigenous people in Ontario, 71% of the participants who attempted to obtain addiction treatment services were unable to access this service [6]. Because transgender persons are regularly grouped with sexual minorities in research studies there is little available information on the experiences of transgender individuals in residential treatment settings. Given the known and vast differences between transgender and sexual minority populations there is a major gap in the literature examining the treatment experiences of transgender populations that we seek to address herein.

## Methods

Between June 2012 and May 2013 the first author conducted in-depth semi-structured interviews with 34 transgender individuals engaged in drug use and/or sex work. Participants were recruited from three open prospective cohorts of individuals who use drugs and an open prospective cohort of sex workers. In addition, three participants were referred to the study by other participants. Eligibility included a) identifying as a person whose gender identity or expression differs from their assigned sex at birth, b) having exchanged sex for money or having used illicit drugs, c) residing in the Greater Vancouver area, and d) being 14 years of age or older. The interview guide, which was guided by an extensive literature review on transgender populations and health, sex work, substance use, was comprised of ten topics (e.g., addiction treatment, housing, access to health services) to capture a range of experience given the dearth of literature on the lived experiences of transgender persons in the drug use and sex work settings under study. The interviews lasted approximately one hour and were conducted at research offices in Vancouver, Canada. No participants declined to be interviewed or left the study and participants were paid $20 to compensate for their time. All of the interviews were audio recorded with permission and every participant provided written informed consent. This study has received annual ethical approval through Providence Health Care/University of British Columbia Research Ethics Board and pseudonyms are used to protect the identity of participants.

### Analysis

All interviews were transcribed verbatim and imported into ATLAS.ti (version 7), a qualitative data management software. Theoretical thematic analysis [17] in conjunction with research questions guided the first-level coding. Two transgender participants were hired as research assistants to conduct the second- and third- level analyses with the first author in a process they developed, called participatory analysis. At each participatory analysis session the data associated with a first-level code (e.g., stigma) was printed and divided into 2 sections, with one half analyzed independently by each person. As a second step, the sections were traded between the first author and the research assistant in order for each section to be analyzed by each person. We validated the codes, corrected any coding errors, and discussed theoretical approaches. Codes were separated analytically into sub-codes and new codes were pulled out from the analysis using an inductive approach [18]. The 24 one-on-one analysis sessions, which ranged from 2 to 3 hours, were held at research offices. Using a participatory analysis approach enriched and contextualized the research findings and provided an opportunity to engage with research participants beyond an interview setting.

## Results

Of the 34 participants in our study, 14 reported ever attending residential treatment and 20 reported never attending residential treatment. Of the 14 participants who attended residential treatment, all had been assigned male sex at birth; however they described their gender identity in different ways and used different pronouns (e.g., she, they). Nine identified as transgender, 4 identified as two-spirit, and 1 reported dressing as a woman in the context of sex work. Two-spirit is a translation of a Northern Algonquin term used to describe an indigenous person who has feminine and masculine spirits [19]. Two-spirit is a fluid, non-binary term and as such it is used by some indigenous people to describe their sexual orientation as lesbian, gay, bisexual, or queer [20]. Participants ranged in age from 27 to 47 years of age, with an average age of 36.8 years. Thirteen participants identified as Indigenous (First Nations or Métis) and 1 identified as Caucasian. Just over half of the participants (*n* = 8) reported currently using illicit drugs (e.g., heroin, crystal methamphetamine, cocaine) excluding cannabis, 3 reported only consuming cannabis, and 3 reported no current drug use, but had a history of drug use. Seven participants reported attending men’s-only treatment facilities, while 4 reported attending women’s-only and 3 reported attending mixed gender facilities. Below we present three themes that characterized transgender individuals’ experiences in residential treatment settings.

### Social rejection, harassment, and violence as enacted stigma

Participants in our study experienced enacted stigma, defined as incidents of discrimination (e.g., rejection, lack of support, denial of service, violence) [15] in treatment settings. Those who reported negative encounters described enacted stigma ranging from name-calling to violence by other residents in treatment settings. For example, Eva attended a mixed gender treatment facility and because she was placed within a women’s section, she had limited contact with men. Despite this separation, she experienced harassment by men in the treatment setting:

I think some men did [make an issue of my gender] to try to be mean to me but, I just wouldn’t have any of it. I’d be like get lost.

Participants also described social rejection and harassment. For example, Marion discussed having conflicts with others in treatment about their gender identity and stated, “I just didn’t know where I belonged”. Julia noted being targeted by others in the treatment setting, which resulted in her isolating herself from others and leaving treatment after a week.

I had a lot of support from the staff, but with the other clients, it was really difficult. I mean everybody’s talking in the whole unit about me. …‘cause I’m the only transgender. … I ended up isolating myself and locked in my room 24 hours a day. … It’s like this isn’t dealing with your addiction.

Reports of enacted stigma from staff were less common; however, participants discussed staff not understanding their gender identity. For example, when Julia arrived at the treatment centre there was confusion about her gender identity:

It was really difficult. I went there and … when I got there they had no idea I was transgender. … They didn’t know how to deal with it.

Casey described conflicts with their treatment counsellor:

[My counsellor] said that I wasn’t being true to myself because I was not acting like a normal two-spirited person would and I would argue with her like, well not argue, but debate with her, how am I supposed to act. Am I supposed to stay here and pop a hip every time? [She was saying you weren’t feminine enough?]. Yeah, it was weird. I just didn’t really like talking about it and … she was rude. She was really, really rude. So yeah, I left.

Casey’s counselor made comments about how feminine they should be acting and engaged in debates with them about their gender presentation. This resulted in Casey feeling uncomfortable and judged, and subsequently they left treatment prematurely.

Physical and sexual violence were other forms of enacted stigma that participants reported. Leah described her experience in a mixed gender facility:

There was a guy that threatened me in there and told me he was gonna kill me. He was calling me a faggot and it was brought to the staff. … So the staff and me and the guy all sat down and they still kept the guy on the unit. I left because I felt unsafe there.

There were also reports of sexual violence in our study. For example, Riley describes an experience in a men’s treatment centre:

Buddy’s sitting there constantly walking by and rubbing his genitals… He’d sit there, rub his cock. He’s like, hey fudge packer come over and sit on my cock.

This encounter escalated into a physical fight and the staff attempted to expel Riley from treatment. After a meeting with the director of the facility, the other client was removed from the treatment program. The director also responded by stating homophobic and transphobic comments would no longer be tolerated and they would be considered punishable acts. Riley noted the director of the facility saying, “all the gay men and trans that have been through here, all you boys made it hard for them and they’ve all left because of your ignorance”.

These two experiences demonstrate the importance of staff interventions in violence. Leah continued to feel unsafe after staff intervened and consequently left treatment, while Riley had support from staff and continued on with treatment. As these examples illustrate, participants encountered various forms of enacted stigma from staff and other individuals in the treatment setting and many of the participants who experienced enacted stigma in treatment settings also reported leaving treatment prematurely.

### Transphobia & felt stigma

Felt stigma captures the fear of experiencing some form of enacted stigma, [15] and is another form of stigma that characterized participants’ treatment experiences. Felt stigma was evidenced in participants’ beliefs that their presence was a disturbance to others in treatment. Rachel explained why she had not attended treatment in the past:

It’s kinda hard to go in there as a transgender person because all the energy and all the focus would be on that…Someone will say you’re a diversion from the rest of the class.

Additionally, Taylor expressed fear of being judged by other individuals in treatment.

In the groups I wouldn’t come out and talk about certain things because there were other straight people there. But I know that lesbian and gay people and trans, if they heard me talk about these other things they wouldn’t go ‘oh my god’, but a straight person would.

Felt stigma helps explain how participants internalized fears of experiencing transphobia in treatment settings. The fear of being a nuisance or diversion from others in the treatment program resulted in not accessing treatment or limiting what they shared in treatment groups.

The common threads through the above examples are participants’ feelings of being unwelcomed, isolated, and unsafe in residential treatment settings. Participants reported not having their treatment needs met as well as prematurely leaving treatment after experiences of enacted and felt stigma. As one of the researcher assistants who conducted the participatory analysis noted: “This leads to us to feeling like we have no right to exist. We are seen as a distraction to other people, as a disturbance, which puts others’ needs before our needs”. In contrast, some participants reported having positive experiences in treatment settings, which we turn to next.

### “Trans friendly” and inclusive treatment experiences

Participants who reported positive treatment experiences reported being accepted and having their gender identity respected by staff and others in treatment settings. For example, Amelia reported that the staff and other clients at a women’s only treatment centre “accepted me…they didn’t judge me”. Additionally, two participants recounted “trans friendly” experiences in indigenous treatment settings. Rielle explained what made her treatment experiences unique:

Their staff was knowledgeable around trans people, the terminology… I was put in on a women’s side as I requested and when it came time to doing the groups I was obviously put with the women versus put with the men. … I did the sweats with the women. I did everything that the women did, I was included in that and I wasn’t excluded from that or anything. I shared a room with another female and it was good.

Rielle described participating in all aspects of treatment as a woman in the indigenous treatment settings. In general, those who remained in treatment and/or recounted positive treatment experiences reported being included in treatment settings by having their gender identity respected and being placed with the appropriate gender in treatment groups and housing.

## Discussion

As part of a larger qualitative study on the experiences of transgender individuals who use drug and/or engage in sex work, this paper has outlined the addiction treatment experiences of transgender individuals in a Canadian setting. The findings illustrate how stigma works to exclude transgender persons from treatment settings. Specifically, many transgender individuals in our study did not have their treatment needs met due to enacted and felt stigma In addition, we found that participants who reported positive treatment experiences had received treatment within settings that understood and respected transgender persons. Thus, our findings demonstrate the importance of fostering respect and inclusivity of gender diverse individuals in residential treatment settings.

This study is one of a very small number that explores the experiences of transgender individuals in a treatment setting. Because transgender populations are often excluded from research or grouped with sexual minorities (e.g., gay men), this study presents a starting point for more in-depth research into how to decrease stigma and transphobia in addiction treatment. The experiences of felt and enacted stigma in treatment settings are supported by the few studies examining treatment experiences of transgender individuals. For example, Senreich [16] found transgender participants in mixed gender treatment facilities felt lower levels of support and connection while in treatment and they were less likely to complete the treatment program compared to heterosexual, gay and bisexual counterparts. Likewise, in Namaste’s [21] study transgender persons reported feeling isolated at treatment centres. We were unable to locate studies that illustrated positive treatment experiences for transgender persons and therefore our findings may indicate an important direction for future research, and more importantly directions for program development.

Indigenous peoples were vastly overrepresented in our study and this is explained in part by our sampling methods where participants were sampled from cohorts of people who use drugs and a cohort of sex workers in an area characterized by disenfranchisement and social inequalities. Indigenous persons are overrepresented in the local environment due to colonialism and the displacement of indigenous people in Canada [22]. Two-spirit people have reported moving to urban areas after facing homophobia and transphobia [20,23] and as such may be further overrepresented in our urban study setting. Historically, two-spirit people were included in their communities and often they held high social status and roles in ceremony. Colonialism and the ongoing attempts by the state to destroy indigenous peoples and their cultures includes practices such as residential schools, forcibly removing indigenous children from homes, displacement of land, and violence [24-26]. The legacies of colonization are inseparable from the current health inequities and discrimination which burden many indigenous peoples [27]; legacies which are evident in our study sample of transgender individuals.

There is a debate in the literature regarding whether specialized treatment settings should be established for LGBTQ groups or whether treatment staff and programs should be better tailored to the needs of the LGBTQ individuals across treatment settings [28-30]. In our study, participants advocated for the development of transgender and/or LBGT combined treatment programs, recovery houses, and treatment centres. The desire for transgender specific treatment programs was driven by wanting a place where participants felt they belonged and where they were supported and accepted. While such places may take time to develop, changes can be made to existing programs to ensure an inclusive and a supportive therapeutic environment for transgender individuals, such as hiring transgender staff, transgender-related training of staff, implementing policies to prevent discrimination and violence, and establishing and modeling guidelines of respect.

Residential treatment programs, transgender specific or otherwise, are not a single solution to substance use among transgender populations. Treatment programs alone cannot address economic, gender and socio-structural disenfranchisement that burdens many transgender persons. To improve the health and treatment outcomes of transgender populations, including those who use drugs, it is imperative to design and evaluate interventions and policies that seek to support participation in the workforce, access to transition-related healthcare for those interested in transition, and anti-stigma education and policies (e.g., introducing gender identity education and polices in schools). One example is Argentina’s Gender Identity Law (Law 26,743 24/05/2012) which was established in May 2012 [31]. This law is based on a framework that affirms equity and human rights, the right to self-defined gender identity, and allows for changes to gender, image, or birth name on their identity card and birth certificates without any requirement of psychiatric evaluation [32]. The law also recommends universal coverage for transition-related healthcare; however, the impact of this law, and others like it, remains under-evaluated.

There is pronounced heterogeneity of transgender populations and as such the study sample cannot be assumed to represent all gender diverse individuals. In particular, it is important to note that the participants were sampled from cohorts of individuals who use drugs and a cohort of sex workers and therefore the findings may not be generalizable to other transgender populations. Future research would benefit from a focus on young transgender persons as they may have unique experiences seeking addiction treatment. Additionally, including two-spirit and transgender participants in the sample may overshadow the unique experiences of two-spirit individuals. Future research would benefit from two-spirit specific research conducted by indigenous peoples and/or in accordance with indigenous research methods and ethics. Lastly, our data was based on self-report and may be susceptible to response biases.

In conclusion, this study highlights the urgent need to implement policies and practices to ensure transgender individuals experience inclusivity and have their gender identity respected in treatment settings. Such changes may improve treatment outcomes, and we suggest evaluations of transgender-inclusive policies and treatment settings are important areas of future research. Our study gives examples of how stigma is a socially embedded process that contributes to social inequalities, such as access to treatment programs. Thus, it is vital that in addition to establishing anti-stigma policies and practices within treatment settings, broader anti-stigma research and activism is undertaken to combat the discrimination and harassment that many transgender groups are burdened with in their daily lives.
